# Multidisciplinary Care in a Public University Family Medicine Group in Québec (Canada): Data on Patients’ Follow-Up and Cardiometabolic Risk Management

**DOI:** 10.3390/healthcare13141704

**Published:** 2025-07-15

**Authors:** Lise Leblay, Léanne Day Pelland, Josée Gagnon, Valérie Guay, Sophie Desroches, Jean-Philippe Drouin-Chartier, Jean-Sébastien Paquette

**Affiliations:** 1 Centre Nutrition, Santé et Société (NUTRISS), Institut sur la Nutrition et les Aliments Fonctionnels (INAF), Université Laval, Québec, QC G1V 0A6, Canada; 2 Faculté de Pharmacie, Université Laval, Québec, QC G1V 0A6, Canada; 3 Faculté de Médecine, Université Laval, Québec, QC G1V 0A6, Canada; 4 Groupe de Médecine de Famille Universitaire du Nord de Lanaudière, CISSS Lanaudière, Saint-Charles-Borromée, Québec, QC J6E 0W6, Canada; 5 École de Nutrition, Faculté des Sciences de l’agriculture et de l’alimentation, Université Laval, Québec, QC G1V 0A6, Canada; 6 Département de Médecine Familiale et de Médecine d’urgence, Faculté de Médecine, Université Laval, Québec, QC G1V 0A6, Canada; 7 VITAM, Centre de Recherche en Santé Durable, Université Laval, Québec, QC G1J 2G1, Canada

**Keywords:** multidisciplinary care, cardiometabolic risk, research-oriented clinic

## Abstract

**Background/Objectives:** Generating real-world data on the efficacy of multidisciplinary care in cardiometabolic risk management is essential to ensure that guidelines are both applicable and effective, especially in public healthcare settings, where organizational structures may impede healthcare professionals’ agility. This study aimed to generate data on patient follow-up and cardiometabolic risk management during the early years of a public university family medicine group in Québec (Canada) that provides multidisciplinary care to adults with cardiometabolic conditions, in order to evaluate the implementation and effectiveness of its care model. **Methods:** This was a retrospective longitudinal study. Patients treated at the clinic from 31 January 2020 (clinic opening) to 8 May 2024 (n = 96) were invited to consent to the use of their medical data for research. **Results:** A total of 52 patients consented and were included in the study. Upon entry at the clinic, >90% of patients had anthropometry and blood pressure (BP) measured, but plasma glucose and lipids were assessed among 50% and 79% of patients, respectively. A total of 36 patients completed the personalized multidisciplinary care program. No evidence of associations between the total number of appointments or appointments with the registered dietitian specifically with changes in BMI, waist circumference, and BP was found. However, each pharmaceutical intervention was associated with a −0.51 cm (95%CI: −1.03, 0.02; *p* = 0.06) change in waist circumference and a −1.49 mm Hg (95%CI: −2.56, −0.43, *p* = 0.01) change in diastolic BP. **Conclusions**: These data highlight the challenges of implementing a research-oriented clinic within Québec’s public healthcare system.

## 1. Introduction

According to the World Health Organization, cardiovascular diseases (CVDs) accounted for 17.9 million deaths in 2019, representing 32% of global mortality [1]. CVDs remain the leading cause of death worldwide [1], and the second leading cause in Canada [2,3,4]. The risk of developing CVD increases due to numerous cardiometabolic risk factors [5,6], including atherogenic dyslipidemia, hypertension, and insulin resistance which can progress to type 2 diabetes [2,7]. Effective management of these risk factors through healthy lifestyle habits, particularly dietary improvements, alongside appropriate medication is essential for reducing CVD risk [6].

Our research group recently highlighted a lack of complementarity between diet quality and medication use in the management of dyslipidemia, hypertension and type 2 diabetes among adults from the Province of Québec, Canada, raising questions on the quality of overall cardiometabolic risk management in this population [8,9,10]. Specifically, we observed that among individuals with metabolic syndrome, statin use was associated with lower diet quality [10]. Similarly, in young adults with hypertension or type 2 diabetes, the intensity of blood pressure (BP)- and glucose-lowering medications was inversely related to diet quality [8,9]. Likewise, we observed that adherence to healthy dietary principles negatively correlates with adherence to cardiopreventive medication among men [11]. Finally, in all three studies, diet quality was highly suboptimal, independent of medication use [8,9,10]. These findings may, in part, stem from insufficient access to nutrition counseling from healthcare professionals. Indeed, previous studies on primary care practices in Canada indicated that individuals with cardiometabolic risk factors often receive limited dietary guidance from their physicians [12,13]. On the other hand, multidisciplinary approaches that integrates physicians, registered dietitians (RD), nurses, and other healthcare professionals are increasingly recognized to enhance the efficacy of lifestyle interventions, also translating into improved cardiometabolic risk management [14,15]. Still, real-world data on the effectiveness of such framework within Quebec’s public healthcare system are crucial to ensuring that clinical recommendations for cardiometabolic risk management are both applicable and effective. In fact, several challenges related to the multidisciplinary approach *per se* have already been identified, including patients’ lack of time, poor communication between health professionals, and limited availability of financial and human resources [16,17,18]. This is particularly relevant given the challenges faced in implementing a research-oriented clinic, including research planning, lack of training for health professionals, fear of negative impacts on the healthcare teams and patients, productivity concerns, the lack of technological tools, limited access to funding resources, and a shortage of human resources [19,20]. Given the challenges associated with the multidisciplinary approach and the integration of research into clinical practice, it is necessary to assess whether such an approach is effective for cardiometabolic risk management in this context.

This study used clinical data from the early years of a public university–family medicine group (U-FMG) in Québec (Canada), which provides personalized multidisciplinary care to adults with cardiometabolic conditions, to generate data on patient follow-up and cardiometabolic risk management, in order to evaluate the implementation and effectiveness of its care model. Overall, this study highlights the challenges of implementing a research-oriented clinic within Québec’s public healthcare system.

## 2. Methods

The study protocol was reviewed and approved by the Université Laval Research Ethics Committee, and the Research Ethics Committee of the *Centre intégré de santé et de services sociaux de Lanaudière*.

### 2.1. Study Population

This study is a retrospective longitudinal analysis using data from the Méta-Santé clinic affiliated with the U-FMG of Northern Lanaudière. The Méta-Santé clinic, located in the city of Joliette (Province of Québec, Canada) was established in January of 2020 provides personalized prevention, treatment, and follow-up services for obesity and associated cardiometabolic conditions (e.g., hypertension, dyslipidemia, type 2 diabetes). To access the clinic’s services, individuals aged over 18 years must be referred by a healthcare professional from the U-FMG and have obesity, defined as a body mass index (BMI) greater than 30 kg/m^2^. The clinic’s team includes a medical doctor, a nurse practitioner, a clinical nurse, a RD, a kinesiologist, and a pharmacist. If needed, patients could also consult with a social worker and a physical therapist.

**Figure 1:**
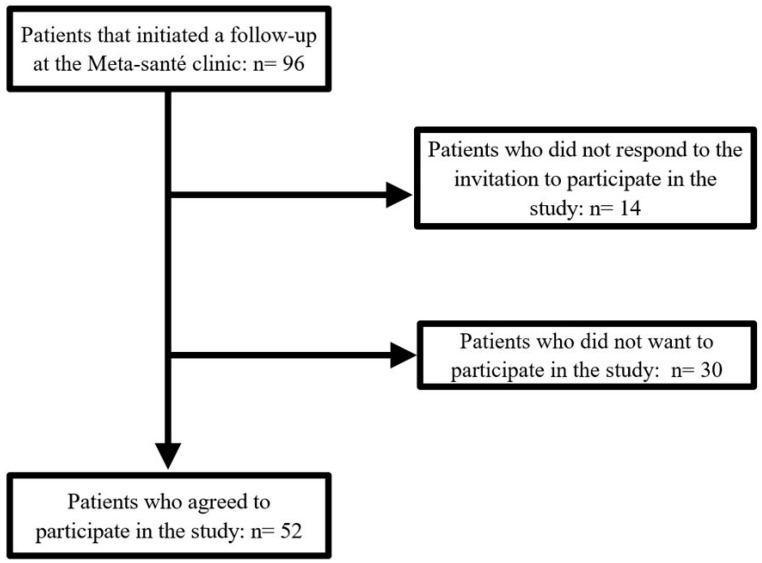
Flow chart of patients from the Meta-Santé clinic followed between 31 January 2020 and 8 May 2024.

For this study, individuals treated at the clinic between 31 January 2020 (clinic opening) and 8 May 2024 (n = 96) were contacted from January 2024 by a member of the research team (LL) and invited to consent to the use of their medical data for research purposes. A total of 52 patients provided consent and were included in the study (Figure 1).

### 2.2. Clinic Follow-Up

The Meta-Santé clinic provides personalized multidisciplinary care. In the current context, personalized multidisciplinary care refers to a coordinated intervention in which patients received individualized care plans developed jointly by a team composed of a medical doctor (physician), nurse practitioner, registered dietitian, clinical nurse, kinesiologist and pharmacist. Care was tailored to each patient’s clinical profile, health goals, and lifestyle, with the frequency and content of follow-up visits adjusted accordingly.

The planned clinic follow-up included the following: At their entry to the clinic, patients were required to attend a one-time group session led by the clinical nurse, RD, and kinesiologist. This session focused on setting specific, measurable, achievable, relevant, and time-bound goals and aimed to inform patients on healthy lifestyle habits, healthy eating, and nutrition labels. At the end of the session, patients officially registered with the clinic. Between 27 March 2020 and 3 December 2021, and again between 21 January 2022 and 8 April 2022, because of the COVID-19 pandemic, virtual classes replaced in-person sessions.

Once registered, each patient was scheduled for an initial individual consultation with the nurse practitioner, the clinical nurse, the RD, and the kinesiologist. The clinical nurse was responsible for collecting sociodemographic information (e.g., age, sex), evaluating lifestyle habits (e.g., alcohol consumption, smoking status, illicit drug use), and monitoring physical health by measuring height, waist circumference, weight, and BP. The nurse practitioner assessed comorbidities (e.g., type 2 diabetes, hypertension, and dyslipidemia), reviewed biochemical risk factors, and adjusted medications as needed. The RD evaluated dietary habits and provided nutrition counseling, while the kinesiologist evaluated physical activity practice and offered tailored recommendations. When necessary, the medical doctor would provide care and prescribed or adjusted medications. The pharmacist’s role was to review pharmacotherapy upon request from other members of the team. Follow-up visits—including their frequency and the clinic professionals involved—were individualized based on each patient’s needs and condition. The maximum follow-up duration at Méta-Santé was 12 months, after which patients were referred back to their regular family medicine clinic. A patient was considered to have dropped out of the clinic if there was no indication in the electronic medical record that the clinic team, in agreement with the patient, had decided to end the follow-up.

### 2.3. Collection of Clinical Data

Height was measured using a stadiometer designed for adults, while weight was assessed with an electronic scale. Waist circumference was recorded using a measuring tape. BMI was calculated based on height and weight measurements. Height and waist circumference were to be measured upon entry into the clinic and then every three months.

Systolic and diastolic BP were measured using an electronic BP monitor (73MT-B Connex Spot Monitor, Welch Allyn, NY, United States) with an appropriately sized cuff, positioned one inch above the elbow crease. Measurements were taken after five minutes of rest in a seated position, with legs uncrossed, back supported, without speaking or moving, with the arm properly supported and feet flat on the floor. If BP values did not meet the target range, an in-clinic assessment was performed using serial oscillometric monitoring. BP was to be measured upon entry into the clinic and then every three months but may be taken more frequently depending on the patient’s condition. 

Every three months during the clinical follow-up, patients were invited to complete three consecutive self-reported daily food diaries using the Keenoa application (version 1.0.3). This mobile app was developed and validated by RDs to evaluate dietary intake among Canadian adults [21,22]. It functions as a food diary, allowing users to photograph their meals and snacks, which are then identified by an artificial intelligence-based algorithm [22]. If a food item is not recognized, then users can manually search for it among the 5690 foods listed in the 2015 Canadian Nutrient File [21]. If the item is not listed, users can describe it. Portion sizes are estimated using visual aids, and grocery item barcodes can also be scanned [22]. The application provides RD with access to the data and a detailed nutritional analysis [21,22]. These data were to be used by the RDs to provide individualized dietary counseling.

Biochemical parameters, including plasma lipids and markers of glucose homeostasis, were obtained from blood tests conducted outside the clinic. Upon entry into the clinic, the medical doctor prescribed blood tests if they had not been performed within the past year. Thereafter, blood tests were prescribed based on individual needs.

### 2.4. Data Extraction and Computation

Among patients who provided consent to the use of their medical data for research purposes, the clinical data were extracted from electronic medical records, when available. Data collection was performed through double entry by two members of the research team (LL and LDP).

Before the analyses, the following computations were made. The follow-up duration was calculated as the number of days between the patient’s first visit to the clinic and their last recorded appointment. The total number of follow-ups corresponded to the sum of all completed visits, with each encounter with a healthcare professional considered a visit. The number of pharmaceutical interventions refers to the total number of pharmacotherapy adjustments made during follow-up. These adjustments could include the prescription of a new medication, deprescription, a dose increase or decrease, or the substitution of one medication for another. Changes in cardiometabolic risk factors (weight, waist circumference, BMI, systolic and diastolic BP, plasma lipids) were calculated as the difference between the first and last available measurement for each participant. These changes were calculated only for participants with at least two available measurements.

### 2.5. Statistical Analyses

Statistical analyses were performed using SAS Studio software (version 3.5). All statistical tests were two-sided with a significance threshold set at *p* < 0.05.

We first used descriptive statistics to summarize patients’ sociodemographic and clinical profiles. We also characterized the type, availability, and completeness of clinical data extracted from the electronic medical record for the purpose of this study using descriptive statistics.

Next, we compared characteristics between patients who completed clinical follow-up and those who dropped out. Separate models were used for each variable. For continuous variables, Student’s t-tests were used to compare means. For categorical variables, chi-square tests were used to compare proportions.

We then used linear mixed models (PROC MIXED) to analyze the association between changes in cardiometabolic risk factors (i.e., weight, waist circumference, BMI, and systolic and diastolic BP, treated as continuous dependent variables) and follow-up intensity (i.e., total number of follow-ups, number of follow-ups with the RD, and number of pharmaceutical interventions—independent variables). Beta coefficients and 95% confidence intervals were calculated for each outcome. Three different models were used. Model 1 was not adjusted. Model 2 was adjusted for age, sex (male/female), baseline smoking status (never, former, current), and baseline alcohol consumption frequency (never, monthly, weekly, daily). Model 3 was adjusted for age, sex (male/female), baseline smoking status (never, former, current), baseline alcohol consumption frequency (never, monthly, weekly, daily) and BMI at baseline (kg/m^2^). For outcomes related to weight, waist circumference, and BMI, Models 2 and 3 were also adjusted for weight loss medication use. For systolic and diastolic BP outcomes, Models 2 and 3 were adjusted for antihypertensive medication use. In models using the number of follow-ups (total and with the RD) as independent variables, weight loss, or antihypertensive medication use was categorized as none; used from baseline through follow-up; initiated after baseline. In models using the number of pharmaceutical interventions as the independent variable, medication use was categorized as used at baseline (yes/no), because any change in medication after entry was considered as a pharmaceutical intervention.

For all statistical models, normality was assessed by examining the distribution of scaled residual values. If residuals were not normally distributed, the Box–Cox transformation (TRANSREG procedure) was applied to identify an appropriate transformation. If normalization was not achieved, a rank transformation (RANK procedure) was used. To facilitate interpretation of models requiring transformation, tables present untransformed β coefficients and confidence intervals, with *p*-values calculated from the transformed model.

## 3. Results

Characteristics of the 52 individuals included in the study are presented in Table 1. Participants had a mean age of 50.3 ± 15.2 years and were predominantly female. Most participants had no personal history of cancer or CVD, and about 1 in 4 had type 2 diabetes. About 40% had high BP, and among them, 81% were receiving antihypertensive medication. About 28% of participants had elevated blood cholesterol levels, with over two thirds taking lipid-lowering medication. The mean waist circumference was 126 ± 17 cm, the mean BMI was 42.1 ± 8.1 kg/m^2^, and the mean triglyceride levels were 2.45 ± 2.81 mmol/L.

The data available for research are presented in Table 2. Among the 52 participants, over 90% had anthropometric and BP measurements at baseline, but only half had at least two measurements during follow-up. Plasma glucose and lipid profiles were assessed at baseline in 50% and 79% of participants, respectively, but only 10% and 25% had at least two measurements during follow-up. Dietary intake was assessed in only 54% of participants at the entry, with just 17% having at least two dietary assessments during follow-up. The rates were slightly higher in the sample consisting only of participants who completed their follow-up, except for biochemical data at baseline.

A total of 36 out of the 52 participants included in the study completed the personalized multidisciplinary care program (Table 3). Participants who dropped out (n = 16) tended to be more likely to be male (*p* = 0.07) and to have high BP (*p* = 0.12), high blood cholesterol (*p* = 0.03), and a greater body weight (*p* = 0.11). Those who completed the program had a longer follow-up duration (*p* < 0.0001), as well as a higher number of total appointments (*p* = 0.002), appointments with the RD (*p* = 0.01), and appointments with the clinical nurse (*p* = 0.01). In terms of pharmaceutical interventions, among those who completed the program, the most frequent changes involved dosage modifications, particularly for glucose-lowering drugs (Supplemental Table S1).

Due to the limited sample of patients with repeated diet data (n = 9), we could not evaluate changes in diet over follow-up. Still, we assessed whether the number of appointments at the clinic, including those with the RD, correlated with changes in cardiometabolic risk factors. No evidence of associations was found between the total number of follow-ups or follow-ups with the RD and changes in BMI, waist circumference, or BP (Table 4). However, each pharmaceutical intervention was associated with a −0.51 cm (95% confidence internal (CI): −1.03, 0.02; *p* = 0.06) change in waist circumference, and a −1.49 mm Hg (95% CI: −2.56, −0.43, *p* = 0.01) change in diastolic BP.

## 4. Discussion

This study aimed to generate data to evaluate patients’ follow-up and cardiometabolic risk management in a public U-FMG clinic providing multidisciplinary care to adults with cardiometabolic conditions in Québec (Canada). Our findings revealed important issues in clinic procedures that hindered the clinic’s ability to collect data for research purposes and to demonstrate the effectiveness of the implemented multidisciplinary model in managing cardiometabolic conditions. For example, beyond anthropometric and BP measurements, baseline data collection was problematic for both biochemical analyses and dietary assessments. At follow-up, missing data were even more prevalent and included anthropometric measurements as well. Additionally, 30% of patients who initially consented to participate in research dropped out of the program before its completion. These factors significantly limited our statistical power to link the multidisciplinary follow-up with improvements in cardiometabolic risk factors. Overall, these findings underscore the challenges of implementing a research-oriented clinic within Québec’s public healthcare system.

Of the 96 patients contacted, only 52 agreed to participate in the study. Comparing characteristics of participants and non-participants would have been very informative for identifying potential barriers and facilitators to research participation. However, due to ethical constraints, we were unable to conduct this comparison. Still, these data suggest the presence of barriers to patient participation in research. In that regard, a cross-sectional study assessing adults’ preferences regarding consent procedures in medical research reported that 84.5% of respondents preferred to be approached for consent by a physician involved in their care, rather than by researchers external to the clinical team [23]. However, due to the absence of a standard consent appraisal procedure upon entry at the Méta-Santé clinic, the research team, independent of the clinic staff, had to contact patients directly, which may have limited participation. Beyond that, data security concerns have been identified in several studies as a significant deterrent to research involvement [24,25,26,27]. For example, a qualitative study found that participants expressed concerns about the sensitive nature of electronic health record data, as well as issues related to confidentiality and privacy in the context of research use [25]. Standardizing the consent process, aligning it with best practices in collaboration with the local ethics committee, and having clinic staff introduce both the overarching goals of the research and the data security protocols may help increase participation rates in future studies at the Méta-Santé clinic.

In addition to the low number of participants included in the study, data collection over follow-up was found to be problematic. Indeed, although anthropometric data and BP were supposed to be assessed every three months, only half of the participants had at least two measurements during the follow-up period. We suggest that issues with physical measurement assessments may be due to the individualized nature of clinical follow-up, which could have disrupted standard procedures and led to missed assessments over time. Therefore, implementing standardized protocols for physical and biochemical assessments during follow-up (e.g., every 3 months) would help facilitate data collection. Similarly, on-site access to blood sampling and biochemical analysis could support the implementation of such protocols.

Only 17% (9 patients out of 52) had at least two dietary assessments during follow-up, even though assessments were scheduled at baseline, and at 3, 6, and 9 months. The limited availability of repeated diet data impeded our capability in assessing changes in diet over follow-up among the patients. Given the relatively high mean number of appointments with the RD, it is unlikely that the lack of data reflects an absence of dietary counseling. Instead, it more likely points to potential barriers to patients’ use of the Keenoa app. A previous qualitative study assessed facilitators and barriers to the use of the Keenoa app [21]. The lengthy process of scanning foods and entering data was identified as a salient barrier to its use. Considering that this qualitative study was conducted among a sample of volunteer participants, we cannot exclude that this barrier is even more important when used in clinical settings among individuals who may have lower interest in tracking their diet to collect data for an upcoming consultation with a RD. Further studies should investigate barriers and facilitators to the use of such an app in clinical settings. In the meanwhile, the Méta-Santé clinic should consider other approaches to collect valid diet data among their patients. For instance, the recently developed and validated Canadian Food Intake Screener [28], which allows to assess adherence to Canada’s food guide within 5 min or less could be an interesting alternative. More broadly, to better understand the challenges experienced by professionals at the Méta-Santé clinic to integrate research into their practice, qualitative research methods, such as semi-structured interviews, could be employed to investigate these [29].

Beyond data collection, maintaining engagement of patients into the personalized multidisciplinary cardiometabolic risk management program appears to also be a potential issue at the Méta-Santé clinic. Indeed, among the 52 participants included in the study, only 36 completed the program. These data suggest that patients faced challenges in adhering to the clinic’s trajectory. Notably, participants who dropped out of this lifestyle-focused multidisciplinary program were more likely to be male, which echoes data from previous studies, suggesting that males may be more tended to downplay the health implications of lifestyle-related risk factors [30]. This finding suggests that males may be less inclined to engage in care programs centered on healthy living. To improve male adherence to the Méta-Santé clinic’s program, one potential strategy could involve developing sex/gender specific trajectories at the clinic. Other factors contributing to dropout from healthcare programs have also been reported in the literature. For instance, lack of time and insufficient social support or staff turnover have been identified as predictors of dropout in a behavioral intervention program for adults with obesity across three primary care centers [31,32,33]. Although specific data on staff turnover at the Méta-Santé clinic were not collected, the clinic operates within Québec’s public healthcare system, which is currently facing a widespread shortage of healthcare professionals [33]. More broadly, ensuring that health professionals use motivational approaches and focus on behavior modification in their interventions could help maximize retention.

The limited data available impeded our ability to draw conclusions about the clinical benefits of the Meta-Santé personalized multidisciplinary care program. Nevertheless, the existing literature supports that such multidisciplinary approaches are generally more effective than standard care. For instance, a comparative study examining the factors that influenced policies on team-based primary healthcare in three Canadian provinces (British Columbia, Alberta, and Saskatchewan) found that multidisciplinary teams improve the quality of care, healthcare professional satisfaction, care coordination, and reduce costs [34]. Also, in the province of Ontario (Canada), integrating nurse practitioners into the Family Health Team model—which is similar to Quebec’s FMG model—allowed physicians to increase their active patient rosters by approximately 800 per year [35]. Whether the addition of registered dietitians and pharmacists can further amplify this effect remains to be documented. However, challenges such as poor communication, financial and human resource constraints can limit the effective implementation of multidisciplinary teams [17,36]. In the Province of Québec, the mission of a U-FMG is three-fold: to provide healthcare, to offer teaching and training to health professional students, and to conduct research. However, the Méta-Santé clinic has received no dedicated financial support for research, meaning that the personalized program and the integration of research procedures were developed with very limited resources. This may help explain the issues identified in this study. To address this, public policies must allocate concrete funding at the local level to support the organization and sustainability of research activities in primary care settings such as U-FMG.

Our results should be interpreted considering certain limitations. The absence of a consent form required us to contact participants directly, which may have limited the number of people willing to participate. Additionally, the electronic medical record platform changed in 2022, which may also have led to information loss; however, this could not be verified. The low number of participants, high dropout rate, and missing data all contributed to the limited statistical power observed in our study. As a result, we were unable to control for comorbidities, medication adherence, or lifestyle changes in the analyses. Despite these challenges, it is important to note that the measurements collected—such as anthropometric data and BP—were obtained according to the recommended guidelines. The study also leverages real-world clinical data and thus provides interesting insights into challenges that could be faced in implementing a research-oriented multidisciplinary clinic within Quebec public healthcare system.

## 5. Conclusions

In this retrospective longitudinal study based on data from the Méta-Santé clinic, a public U-FMG from the Province of Québec (Canada), our findings revealed important issues in clinic procedures that hindered the clinic’s ability to collect data for research purposes and to demonstrate the effectiveness of the implemented model in managing cardiometabolic conditions. These findings underscore the challenges of implementing a research-oriented clinic within Québec’s public healthcare system, despite U-FMG’s mission comprises conducting research, as well as the importance of evaluating barriers and facilitators to research implementation in such settings. Nonetheless, the study also highlighted the added value of collaboration between primary care providers and academic researchers in advancing the quality of interventions in primary care.

## Figures and Tables

**Table 1:** Characteristics of the 52 patients included in the study, at the beginning of their clinical follow-up at the Méta-Santé clinic.

Characteristics	Mean ± SD or N (%)
Age, years	50.3 ± 15.2
Sex	
Female	38 (73.1)
Male	14 (27.0)
Alcohol consumption	
Never	14 (26.9)
Monthly frequency	22 (42.3)
Weekly frequency	12 (23.1)
Daily frequency	4 (7.7)
Smoking status	
Never	28 (53.9)
Former	18 (34.6)
Current	6 (11.5)
Illicit drug use	
Never	49 (94.2)
Former	0
Current	3 (5.8)
Personal history of cancer	
No	48 (92.3)
Yes	4 (7.7)
Personal history of cardiovascular disease	
No	48 (92.3)
Yes	4 (7.7)
Type 2 diabetes	
No	40 (76.9)
Yes	12 (23.1)
High blood pressure	
No	31 (59.6)
Yes	21 (40.4)
Unmedicated	4 (19.0)
Medicated	17 (81.0)
High blood cholesterol	
No	37 (71.2)
Yes	15 (28.9)
Unmedicated	5 (33.3)
Medicated	10 (66.7)
Weight, kg	113 ± 28
Waist circumference, cm ^1^	126 ± 17
Body mass index, kg/m^2^	42.1 ± 8.1
Systolic blood pressure, mm Hg ^1^	130 ± 14
Diastolic blood pressure, mm Hg ^1^	79 ± 10
HbA1c, % ^2^	5.96 ± 0.85
Triglycerides, mmol/L ^3^	2.45 ± 2.81
HDL-cholesterol, mmol/L ^4^	1.09 ± 0.24
LDL-cholesterol, mmol/L ^5^	2.84 ± 1.04

Continuous variables are presented as mean ± standard deviation. Categorical variables are presented as count (percent). ^1^ N = 48/52. ^2^ N = 43/52. ^3^ N = 25/52. ^4^ N = 23/52. ^5^ N = 27/52.

**Table 2:** Data available for research.

Data	All Patients (n = 52)	Patients Who Completed Their Follow-Up (n = 36)
Anthropometry		
Patients with BMI calculated at entry, n (%)	52 (100.0)	36 (100.0)
Patients with BMI calculated at entry and at least 1 time during follow-up, n (%)	30 (57.7)	25 (69.4)
Patients with waist circumference measured at entry	48 (92.3)	34 (94.4)
Patients with waist circumference measured at entry and at least 1 time during follow-up	25 (48.1)	22 (61.1)
Blood pressure		
Patients with blood pressure measured at entry	48 (92.3)	34 (94.4)
Patients with blood pressure measured at entry and at least 1 time during follow-up	26 (50.0)	23 (63.9)
Biochemical analyses		
Patients with a lipid profile measured on or before entry	26 (50.0)	16 (44.4)
Patients with a lipid profile measured on or before entry and at least 1 time during follow-up	5 (9.6)	4 (11.1)
Patients with glucose homeostasis measured on or before entry	41 (78.9)	28 (77.8)
Patients with glucose homeostasis measured on or before entry and at least 1 time during follow-up	13 (25.0)	12 (33.3)
Dietary assessments		
Patients who completed at least 1 dietary diary at entry, n (%)	28 (53.9)	22 (61.1)
Patients who completed dietary diaries at entry and during follow-up, n (%)	9 (17.3)	8 (22.2)

**Table 3:** Comparison of patients who completed the personalized multidisciplinary care program versus those who dropped out.

Characteristics	Patients Who Completed the Program (n = 36)	Patients Who Dropped Out (n = 16)	*p*-Value *
Age, years	50.8 ± 13.9	48.9 ± 18.4	0.72
Sex			0.07
Women	29 (80.6)	9 (56.3)	
Men	7 (19.4)	7 (43.8)	
Alcohol consumption			0.34
Never	10 (27.8)	4 (25.0)	
Monthly frequency	12 (33.3)	10 (62.5)	
Weekly frequency	11 (30.6)	1 (6.3)	
Daily frequency	3 (8.3)	1 (6.3)	
Smoking status			0.23
Never	20 (55.6)	8 (50.0)	
Former	14 (38.9)	4 (25.0)	
Current	2 (5.6)	4 (25.0)	
Illicit drug use			0.17
Never	35 (94.2)	14 (87.5)	
Current	1 (2.8)	2 (12.5)	
Personal history of cancer			0.79
No	33 (91.7)	15 (93.8)	
Yes	3 (8.3)	1 (6.3)	
Personal history of cardiovascular disease			0.79
No	33 (91.7)	15 (93.8)	
Yes	3 (8.3)	1 (6.3)	
Cardiometabolic risk factors			
Type 2 diabetes			0.83
No	28 (77.8)	12 (75.0)	
Yes	8 (22.2)	4 (25.0)	
High blood pressure			0.12
No	24 (66.7)	7 (43.8)	
Yes	12 (33.3)	9 (56.3)	
High blood cholesterol			0.02
No	29 (80.6)	8 (50.0)	
Yes	7 (19.4)	8 (50.0)	
Weight, kg	109 ± 25	123 ± 31	0.11
Waist circumference, cm ^1^	124 ± 15	132 ± 19	0.21
Body mass index, kg/m^2^	41.1 ± 8.0	44.3 ± 7.9	0.18
Systolic blood pressure, mm Hg ^1^	129 ± 12	134 ± 17	0.34
Diastolic blood pressure, mm Hg ^1^	79 ± 8	79 ± 13	0.93
HbA1c, % ^2^	5.88 ± 0.67	6.15 ± 1.17	0.44
Triglycerides, mmol/L ^3^	1.89 ± 0.76	3.17 ± 4.15	0.33
HDL-cholesterol, mmol/L ^4^	1.12 ± 0.20	1.05 ± 0.27	0.49
LDL-cholesterol, mmol/L ^5^	2.74 ± 1.07	2.98 ± 1.04	0.57
Implication in the program			
Attended the baseline group session			0.78
No	15 (41.7)	6 (37.5)	
Yes	21 (58.3)	10 (62.5)	
Duration of follow-up, days	287 ± 90	158 ± 88	<0.0001
Total appointments, n	16 ± 9	9 ± 6	0.002
Appointments with the registered dietitian, n	8 ± 3	5 ± 4	0.007
Appointments with the clinical nurse, n	6 ± 6	3 ± 2	0.01
Appointments with the practician nurse and/or the medical doctor, n	2 ± 1	1 ± 1	0.13
Pharmaceutical interventions, n	1 ± 5	0 ± 1	0.21

* *p*-value from Student’s *t*-test for mean comparisons and chi-square test for proportion comparisons. ^1^ Participants who completed the program: n = 34/36; participants who dropped out: n = 14/16. ^2^ Participants who completed the program: n = 30/36; participants who dropped out: n = 13/16. ^3^ Participants who completed the program: n = 14/36; participants who dropped out: n = 11/16. ^4^ Participants who completed the program: n = 12/36; participants who dropped out: n = 11/16. ^5^ Participants who completed the program: n = 16/36; participants who dropped out: n = 11/16.

**Table 4:** Association between cardiometabolic risk factor changes and the number of appointments and pharmaceutical interventions at the Méta-Santé clinic among the patients who completed the follow-up (n = 36) ^1^.

	β (95% CI) per Each Appointment at the Clinic	*p*-Value	β (95% CI) per Each Appointment with the Registered Dietitian	*p*-Value	β (95% CI) per Each Pharmaceutical Intervention	*p*-Value
Weight, kg (N = 25)						
Model 1	0.08 (−0.15, 0.30)	0.49	0.47 (−0.21, 1.15)	0.17	−0.04 (−0.39, 0.31)	0.82
Model 2	0.04 (−0.35, 0.42)	0.84	0.50 (−0.69, 1.69)	0.38	−0.24 (−0.72, 0.24)	0.30
Model 3	0.03 (−0.37, 0.43)	0.87	0.53 (−0.71, 1.77)	0.37	−0.24 (−0.74, 0.25)	0.31
Waist circumference, cm (N = 24)						
Model 1	−0.33 (−0.64, −0.03)	0.03	−0.92 (−1.89, 0.06)	0.06	−0.66 (−1.09, −0.22)	0.01
Model 2	−0.30 (−0.72, 0.13)	0.15	−0.31 (−1.76, 1.14)	0.65	−0.51 (−1.03, 0.01)	0.05
Model 3	−0.28 (−0.71, 0.15)	0.18	−0.36 (−1.83, 1.11)	0.60	−0.51 (−1.03, 0.02)	0.06
BMI, kg/m^2^ (N = 25)						
Model 1	0.04 (−0.05, 0.13)	0.34	0.16 (−0.11, 0.43)	0.23	0.00 (−0.14, 0.14)	0.87 ^2^
Model 2	0.03 (−0.12, 0.17)	0.72	0.13 (−0.34, 0.60)	0.57	−0.08 (−0.26, 0.11)	0.40
Model 3	0.02 (−0.13, 0.18)	0.76	0.14 (−0.34, 0.62)	0.54	−0.08 (−0.27, 0.12)	0.41
Systolic blood pressure, mm Hg (N = 24)						
Model 1	−0.32 (−1.19, 0.54)	0.44	−0.08 (−2.85, 2.69)	0.95	−0.27 (−1.63, 1.09)	0.68
Model 2	−0.25 (−1.65, 1.16)	0.71	1.51 (−2.33, 5.35)	0.41	−0.55 (−2.66, 1.55)	0.58
Model 3	−0.17 (−1.53, 1.19)	0.80	1.57 (−2.09, 5.24)	0.37	−0.45 (−2.50, 1.61)	0.65
Diastolic blood pressure, mm Hg (N = 24)						
Model 1	−0.24 (−0.69, 0.20)	0.27	0.28 (−1.16, 1.73)	0.69	−0.53 (−1.20, 0.15)	0.12
Model 2	−0.38 (−1.22, 0.46)	0.35	0.43 (−1.99, 2.84)	0.71	−1.47 (−2.51, −0.43)	0.01
Model 3	−0.40 (−1.27, 0.47)	0.34	0.41 (−2.10, 2.92)	0.73	−1.49 (−2.56, −0.43)	0.01

^1^ Data are presented as β coefficients (95% confidence intervals), reflecting the difference in the measured variable associated with each additional appointment (continuous variable). Model 1 was no adjusted. Model 2 was adjusted for age, sex (male/female), baseline smoking status (never, former, current), baseline alcohol consumption frequency (never, monthly, weekly, daily). Model 3 was adjusted for age, sex (male/female), baseline smoking status (never, former, current), baseline alcohol consumption frequency (never, monthly, weekly, daily) and BMI at baseline (kg/m^2^). Models 2 and 3 for weight, waist circumference, and BMI were adjusted for anti-obesity medication use. Models 2 and 3 for systolic and diastolic blood pressure were adjusted for antihypertensive medication use. Models for the number of follow-ups at the clinic and the number of follow-ups with the registered dietitian, categorized medication use as none/at baseline/initiated after clinic entry. Models for the number of pharmaceutical interventions categorized medication used based on their presence at baseline (yes/no). ^2^ Required rank transformation. Untransformed beta coefficients and confidence intervals are presented along with *p*-values calculated from the transformed model.

## Data Availability

Restrictions apply to the dataset. Access is restricted. Requests to access the dataset should be directed to a corresponding author.

## Supplementary Materials

The following supporting information can be downloaded at: https://www.mdpi.com/article/10.3390/healthcare13141704/s1. Table S1: Comparison of the types of pharmaceutical interventions between patients who completed the personalized multidisciplinary care program versus those who dropped out.
