# Supplementary material for: Multidisciplinary Care in a Public University Family Medicine Group in Québec (Canada): Data on Patients’ Follow-Up and Cardiometabolic Risk Management
Source: Healthcare (Basel). 2025 Jul 15;13(14):1704. doi: 10.3390/healthcare13141704 (PMC12294377; doi:10.3390/healthcare13141704)
Supplement: Supplementary file 1 [file healthcare-13-01704-s001.zip › healthcare-3686989-supplementary.pdf]

SUPPLEMENTARY MATERIAL

Table S1. Comparison of the types of pharmaceutical interventions between patients who completed the personalized multidisciplinary care program versus those who dropped out.

	**Patients who completed the program (n = 36)**	**Patients who dropped out (n = 16)**
Participants who initiated a new medication during follow-up, n	12	4
Medication for obesity, n	5	0
Glucose-lowering medication, n	6	1
Cholesterol-lowering medication, n	0	1
Blood-pressure-lowering medication, n	1	2
Participants who stopped a medication during follow-up, n	4	0
Medication for obesity, n	1	0
Glucose-lowering medication, n	2	0
Cholesterol-lowering medication, n	0	0
Blood-pressure-lowering medication, n	1	0
Participants for whom the dosage of a medication was increased during follow-up, n	18	1
Medication for obesity, n	2	0
Glucose-lowering medication, n	16	0
Cholesterol-lowering medication, n	0	0
Blood-pressure-lowering medication, n	0	1
Participants for whom the dosage of a medication was decreased during follow-up, n	23	0
Medication for obesity, n	0	0
Glucose-lowering medication, n	22	0
Cholesterol-lowering medication, n	0	0
Blood-pressure-lowering medication, n	1	0
Participants for whom a medication was replaced by another serving the same purpose during follow-up, n	1	0
Medication for obesity, n	0	0
Glucose-lowering medication, n	1	0
Cholesterol-lowering medication, n	0	0
Blood-pressure-lowering medication, n	0	0
